# Chromatin folding by the Polycomb group proteins and its elusive role in epigenetic repression

**DOI:** 10.1111/febs.70199

**Published:** 2025-07-27

**Authors:** Ludvig Lizana, Yuri B. Schwartz

**Affiliations:** ^1^ Integrated Science Lab, Department of Physics Umeå University Umeå Sweden; ^2^ Epigenetic Cooperation North (EpiCoN), Umeå University Umeå Sweden; ^3^ Department of Molecular Biology Umeå University Umeå Sweden

**Keywords:** chromatin, computational modelling, epigenetics, genome architecture, Polycomb, transcriptional repression

## Abstract

The Polycomb system epigenetically represses selected developmental genes to enforce gene expression programs of differentiated cells. The system requires the coordinated action of dozens of structurally unrelated proteins assembled in two evolutionarily conserved polycomb repressive complexes, PRC1 and PRC2. Genes repressed by the Polycomb system are enriched in histone H3 trimethylated at lysine 27 (H3K27me3), an epigenetic mark that propagates the repressed state after DNA replication. Despite the impressive progress in dissecting molecular functions of the Polycomb group proteins, the fundamental questions of how the Polycomb system represses transcription or how the H3K27me3 mark is translated to benefit the repression are still open. Multiple observations indicate that the binding of PRC1, PRC2, and elevated H3K27me3 correlate with changes in the chromatin structure of target genes, which may be integral for the associated epigenetic repression. In this Review, we summarize our current understanding of these observations. We discuss the chromatin folding inside the loci repressed by the Polycomb system, consider molecular processes causing it and reflect upon its possible impact on transcription and epigenetic memory of the repressed state.

## Graphical abstract

Genes repressed by the Polycomb system display greater chromatin folding compared to ‘regular’ inactive genes. The associated chromatin compaction, driven by PRC1‐H3K27me3 interactions, is modest and dynamic. This questions traditional models where Polycomb proteins repress transcription by sheltering genes from transcriptional activators. How such dynamic chromatin folding helps epigenetic repression or whether it is an ineffectual byproduct of molecular interactions within the Polycomb system are important open questions.

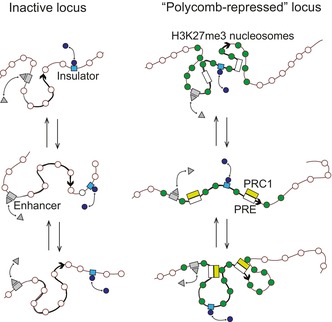

**Abbreviations**
3C: chromatin conformation capture
FISH: fluorescent *in situ* hybridization
GFP: green fluorescent protein
H3K27me3: histone H3 trimethylated at lysine 27
HP1: heterochromatin protein 1
IDR: intrinsically disordered region
LLPS: liquid–liquid phase separation
Mb: million base pairs
O‐GlcNAc: O‐linked N‐acetyl‐glucosamine
PRC: polycomb repressive complex
PRE: polycomb response element
SAM: sterile alpha motif
TAD: topologically associated domain
TBP: TATA binding protein

## Introduction

The Polycomb system is an essential regulatory mechanism to enforce specific gene expression programs when cells differentiate [1, 2]. To this end, the system represses genes for transcriptional regulators and signalling molecules, which would activate alternative gene expression programs and convert the cell into a ‘wrong’ type if accidentally switched on. Importantly, somatic cells transmit the information that a gene has been repressed by the Polycomb system to the daughter cells when they divide. This epigenetic information transfer does not involve changes in the nucleotide sequence of the DNA.

The Polycomb system consists of multiple structurally unrelated proteins, most discovered in genetic screens for repressors of *Drosophila melanogaster* homeotic genes [3, 4, 5]. Biochemical studies demonstrated that these proteins assemble in two kinds of evolutionarily conserved polycomb repressive complexes, PRC1 and PRC2 [6, 7, 8, 9]. We refer the interested reader to recent reviews that describe the composition of PRC1 and PRC2 in detail [2, 10]. Within the cell nucleus, DNA is wrapped around protein particles consisting of positively charged histone proteins (H2A, H2B, H3 and H4). The DNA–histone particles, known as nucleosomes, are critical for the orderly genome folding (for an extended discussion of the genome architecture see: [11]). PRC2 complexes can methylate histone H3 at lysine 27 (H3K27) [7, 12] and one of the PRC1 subunits can specifically interact with histone H3, which carries three methyl groups at lysine 27 (H3K27me3). Both trimethylation of H3K27 by PRC2 and its recognition by PRC1 are required for epigenetic repression by the Polycomb system [13, 14].

In addition to the ‘canonical’ complexes described above, one of the PRC1 subunits (represented by closely related proteins RING1 or RING2) is incorporated in several alternative complexes sometimes referred to as ‘non‐canonical’ or ‘variant’ PRC1 [15, 16, 17, 18]. These alternative RING1/2 complexes lack the subunit that can interact with H3K27me3. PRC1 and alternative RING1/2 complexes can monoubiquitylate histone H2A at Lysine 119 [15, 19, 20]. Multiple lines of evidence indicate that canonical PRC1 is critical for repressing developmental genes [3, 21, 22, 23, 24, 25]. To what extent the monoubiquitylation of H2A or alternative RING complexes contributes to the repression by the Polycomb system is unclear [26, 27, 28, 29, 30, 31]. Therefore, we will keep canonical PRC1 and PRC2 as the primary focus of this review.

Neither canonical PRC1 nor PRC2 incorporates sequence‐specific DNA binding subunits. *Drosophila* genes regulated by the Polycomb system are equipped with specialized short (~1 kb) Polycomb Response Elements (PREs), which are thought to tether PRC1 and PRC2 via multiple sequence‐specific DNA binding ‘adaptor’ proteins. In this view, the adaptor proteins combine their individually weak interactions with PRC1 and PRC2 to retain the complexes or facilitate their association with PREs (for a detailed review of PREs see [32]). The targeting of canonical PRC1 and PRC2 to mammalian genes is less studied, but the emerging picture appears principally similar to that in *Drosophila*. Thus, many human developmental genes contain DNA elements tethering canonical PRC1 and PRC2 [33]. However, in contrast to flies, DNA features associated with PRC1 and PRC2 tethering differ, and the binding of the two complexes to a regulated locus is less strictly linked. Therefore, the human genome contains various DNA elements that range from those that tether primarily PRC1 or PRC2 to ones that can tether both complexes [33].

Despite three decades of fast progress in understanding the molecular workings of the Polycomb system, some basic questions like ‘How does it repress transcription?’ or ‘How is the H3K27 methylation mark translated into transcriptional repression?’ still lack clear answers. It was proposed that H3K27me3 enables the repression by directing the recruitment of PRC1 [34]. However, experimental observations contradict this model. First, genomic mapping shows that PREs or analogous elements in mammalian cells are the sites where PRC1 is stably bound [33, 35], not the H3K27me3‐rich chromatin domains around them. Second, PREs are depleted from nucleosomes and are often the least methylated parts of the repressed genes [35, 36]. Third, PRC1 continues to bind PREs when PRC2 and H3K27 methylation are removed by mutation [37].

The simultaneous binding of PRC1, PRC2 and elevated H3K27 methylation correlates with changes in chromatin structure. It is customarily assumed that such changes antagonize transcription, but the evidence supporting this notion is largely circumstantial. In the following pages, we review our current understanding of the chromatin configuration at loci repressed by the Polycomb system. We attempt to summarize the insights gained from experimental observations and computational modelling, focusing on molecular processes that cause chromatin folding and speculate on possible connections between chromatin folding and epigenetic repression.

## Long‐range interactions

In experiments with immunostaining or GFP‐tagged Polycomb group proteins, it was noted early on that Polycomb group proteins form distinct foci inside *Drosophila* and mammalian nuclei [38, 39]. Sometimes referred to as Polycomb bodies, these foci are fewer than the number of repressed loci detected by genomic mapping. PRE‐mediated repression of reporter genes is noticeably stronger in *Drosophila* with two copies of a transgene [40, 41]. For this so‐called ‘paring‐sensitive silencing’, the two transgenes must be in spatial proximity, which is usually the case in homozygous flies due to the somatic pairing of homologous chromosomes. However, pairing‐sensitive silencing is not limited to transgenic insertions in the same genomic site. It can happen between two transgenes inserted in different chromosomes if, in these transgenes, PREs are coupled to genetic elements that promote *trans*‐interactions [42]. The two notions combined suggest that Polycomb bodies may represent nuclear micro‐compartments where multiple loci, regulated by the Polycomb system, cluster to achieve robust repression. Alternatively, the bodies may correspond to Polycomb complexes bound to multiple tethering elements of the same locus clustered together.

**Fig. 1:**
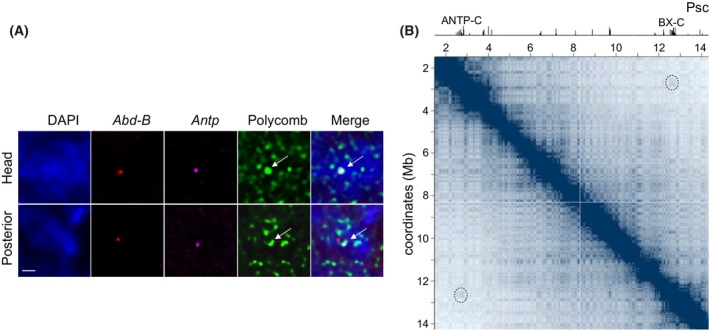
Genes repressed by the Polycomb system are in closer proximity than expected. (A) Combined immunodetection and FISH of Polycomb, *Abd‐B* (the gene from the bithorax complex), and *Antp* (the gene from the Antennapedia complex). Note the three signals colocalizing in the nuclei of the anterior part of the *Drosophila* embryo (head) where the Polycomb system represses both *Abd‐B* and *Antp*, but not in the posterior part of the embryo, where *Antp* is repressed but *Abd‐B* is transcriptionally active. The arrows point to the Polycomb bodies encompassing *Antp* and *AbdB* in the head, but just *Antp* in the posterior part. The scale bars represent 1 μm. The image is adapted from Figure 1D in [44]. (B) Heatmap representation of the chromatin contacts along a segment of *Drosophila* chromosome 3R (dark blue – more contacts, light blue – fewer contacts) [80]. The profile above the heat map shows Chromatin immunoprecipitation sequencing (ChIP‐seq) signals for the PRC1 subunit Psc [37]. Clusters of Psc ChIP‐seq signals mark locations of the Antennapedia (ANTP‐C) and bithorax (BX‐C) complexes. The heatmap shows elevated contact frequency between ANTP‐C and BX‐C (marked with dashed circles). Here and in Fig. 3 the heatmaps were plotted using Juicebox.js [112].

These hypotheses can be tested by simultaneous immunodetection of the Polycomb group proteins and fluorescent *in situ* hybridization (FISH) of DNA probes against the repressed loci or by the chromosome conformation capture (3C) assays. The latter is based on the idea that the spatial proximity of any two genomic fragments can be deduced from the frequency with which these fragments are joined together by the DNA ligase (for an in‐depth review of the approach see: [43]). In the unbiased genome‐wide version of the 3C assay called Hi‐C, live cells are crosslinked by treatment with formaldehyde, their genome digested with a restriction endonuclease and the resulting fragments ligated under conditions favouring the ligation of fragments in close spatial proximity. The fragment junctions are then identified and counted by sequencing. Both approaches concur (Fig. 1) that loci repressed by the Polycomb system tend to be closer to each other in the nuclear space than expected by chance [44, 45, 46, 47, 48, 49, 50]. However, the overall spatial colocalization between distant repressed loci appears to be rare (less than 10% of the time), at least for *Drosophila* where this question was systematically investigated by multiplexed chromatin imaging, and involves predominantly just a pair of genes [51]. In turn, the amount of Polycomb group proteins within specific bodies seems to correlate with the linear size of the incorporated repressed locus and the number of embedded tethering elements (PREs) [52]. These observations argue that most Polycomb bodies inside the nucleus encompass Polycomb complexes bound to multiple tethering elements of a single locus. The low number of Polycomb bodies compared to the number of repressed loci, as identified by genomic mapping, likely stems from the lower sensitivity of microscopy‐based techniques that miss weaker signals emitted by short loci with one or two tethering elements. Infrequently, but with a likelihood greater than expected by chance, single‐locus Polycomb bodies form pairs [51].

What molecular process drives this pairing? Any answer to this question needs to consider the polymer nature of the chromatin [53]. Here computational modelling provides a useful ground framework. Regardless of specific details, most computational models represent chromatin as a linear chain of equally sized monomers corresponding to a short chromatin segment, typically 1–10 nucleosomes long. Therefore, 575 to 5750 monomers are required to simulate the folding of 1 million base pairs (Mb) of chromatin. Applying such a model to entire chromosomes with lengths of more than 10 Mb becomes computationally challenging. This may be solved by increasing the monomer size to include longer chromatin stretches (coarse‐graining). State‐of‐the‐art computational models could be grouped into two primary categories: equilibrium and nonequilibrium [3, 11, 54]. The former focuses on passive binding and unbinding associated with pairwise interactions, such as reversible chemical reactions. These interactions are characterized by binding energies (or dissociation constants) between different polymer sections representing distinct chromatin types (e.g. transcriptionally active genes versus loci repressed by the Polycomb system). In contrast, the nonequilibrium models include active processes that require additional energy (e.g. ATP‐ADP conversion). In such models, the monomers may move due to forces that consume energy and continuously push the system out of equilibrium. The best‐known example of a nonequilibrium model is the chromatin loop extrusion [55, 56].

**Fig. 2:**
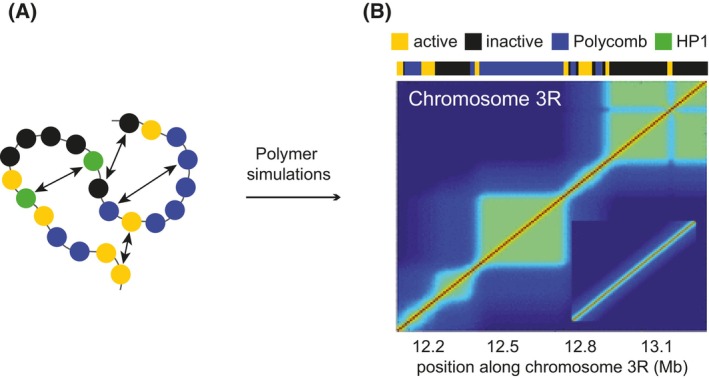
Illustration of the four‐state block copolymer model. (A) Schematic representation of the model by Jost et al. [57]. The chromatin is represented as a self‐avoiding bead‐spring chain where each monomer corresponds to a 10 kb chromatin stretch characterized by its epigenetic state: yellow (active), blue (loci repressed by the Polycomb system), black (transcriptionally inactive), green (loci repressed by HP1). The arrows represent attraction between beads with the same epigenetic state. (B) Simulated Hi‐C contact map for the chromatin region between 12.16 and 13.36 Mb of chromosome 3R. This region lacks HP1 but contains a stretch repressed by the Polycomb system. The positions of chromatin stretches with different epigenetic states are indicated at the top. The inset shows the starting polymer configuration. Adapted from [57].

To date, only equilibrium models have been applied to understand the proximity of loci repressed by the Polycomb system. In one prominent example, Jost and co‐authors designed a block copolymer model that represented four distinct epigenetic chromatin states, one of which corresponded to that of the genes repressed by the Polycomb system [57]. In such a model, a self‐avoiding polymer chain is composed of blocks with different properties that interact with each other according to stipulated rules (Fig. 2). The model faithfully reproduced many general chromatin folding patterns observed in chromosome conformation capture experiments (i.e. Hi‐C). However, it generally did not recover increased proximity between genes repressed by the Polycomb system. To achieve observed spatial proximities, the polymer simulations had to start from a non‐random polymer configuration that mimicked experimental Hi‐C maps. In a more recent attempt, Gurgo and colleagues reported recapitulating experimental proximity frequencies between distant loci repressed by the Polycomb system using a simpler, course‐grained two‐state self‐avoiding block copolymer model [51]. In this model, the authors represented one *Drosophila* chromosome arm as a polymer consisting of 866 beads (20 kb each) with two possible identities: ‘Polycomb’ or ‘not Polycomb’. The distribution of self‐attracting ‘Polycomb’ beads within the simulated polymer mirrored the location of repressed genes along the chromosome arm, while all intervening beads were of the inert ‘not Polycomb’ type. By keeping the attraction between the ‘Polycomb’ monomers as a free parameter, the authors found a range of attraction energies where simulated proximity frequencies matched those detected in the experiment. Of note, at those attraction energies, the polymer reached the folding regime known as the equilibrium globule [58]. A striking feature of this polymer state is that the contact frequency between monomers becomes nearly constant at long distances (>1 Mb), which is at odds with chromatin contact decay profiles measured by Hi‐C [49, 59].

What proteins or molecular interactions may be responsible for attraction between the repressed loci? Two proteins from the Polycomb group were suggested as candidates: Ph (human orthologues PHC1, PHC2, PHC3, component of PRC1) and Scm (human orthologues SCMH1 and SCML2). The *Drosophila* Scm is incorporated as a sub‐stochiometric subunit into both PRC1 and PRC2 [60]. Similarly, mammalian SCMH1 and SCML2 were detected in biochemical purifications of PRC1 [15] and SCML2 was found to co‐immunoprecipitate with EZH2 (core PRC2 subunit) from protein extracts of the germline cell nuclei [61]. Both Ph and Scm contain the sterile alpha motif (SAM) domain that can form homo‐ and heteropolymers *in vitro* [62, 63]. Some authors proposed that the polymerization of Ph drives the formation of the Polycomb bodies [45, 64]. Supporting this hypothesis, the size of the Polycomb bodies in *Drosophila* [64] and mouse cells [45] was reduced after the overexpression of truncated Ph lacking the SAM domain and thus cannot polymerize. However, the SAM domain of Ph is critical for the repression [45, 65], which is consistent with the hypothesis but also complicates the interpretation of the results. In the experiments above, a substantial reduction of the Polycomb body size led to increased transcription of the investigated genes. As transcription is known to unfold the chromatin [66, 67], it is uncertain whether the reported body size changes are mechanistically independent of transcription or are the consequence of it. There are additional confounding factors. Thus, the Ph polymerization appears under strict control *in vivo*. In *Drosophila*, the glycosyltransferase Ogt adds O‐linked N‐Acetyl‐glucosamine (O‐GlcNAc) moieties to Ph, which prevents its aggregation [65]. Impairing O‐GlcNAcylation of Ph by either removing Ogt [68] or truncating Ph [65] impairs the repression. This implies that, in the cell, Ph has a limited ability to polymerize.

In conclusion, we note that both FISH and Hi‐C assays used to study contacts between distant repressed loci rely on chemical crosslinking of live cells. The crosslinking ‘freezes’ all processes inside the nucleus. Therefore, we know very little about the time it takes for two loci to pair or how long the pair exists before drifting apart to find new mates. Emerging live imaging techniques, like those based on CRISPR/Cas9‐mediated locus tagging [69], may help to answer these questions.

## Local chromatin folding

Like many other models explaining the inner workings of the Polycomb system, the popular ideas regarding its impact on the chromatin structure of individual repressed genes emerged from studies in *Drosophila*. The cloning of the *Polycomb* gene and the discovery that its eponymous product (the fly ortholog of mammalian CBX2, CBX4, CBX6 CBX7 and CBX8 proteins) contains the structural motif called Chromodomain shared with the Heterochromatin Protein 1 (HP1) led to the idea that the Polycomb and HP1 systems repress genes via a common mechanism [70]. Strengthening the apparent similarity, transgenes integrated into the vicinity of genes repressed by the Polycomb systems or transgenes equipped with polycomb response elements (PREs) display stochastic inactivation of reporter genes [41, 42]. This resembles the variegated silencing of genes brought by chromosomal rearrangements in the vicinity of HP1‐bound pericentromeric regions [71]. The HP1‐bound regions appear compact in the diploid chromosomes of embryonic cells [72] and in giant polytene chromosomes of salivary glands [73]. Likewise, on polytene chromosomes, the homeotic gene clusters repressed by the Polycomb system appear shrunk and compact. These observations combined led to a view that HP1 and Polycomb group proteins repress transcription by compacting the chromatin of a target gene to a point where it becomes inaccessible to transcriptional activators and transcriptional machinery [74].

**Fig. 3:**
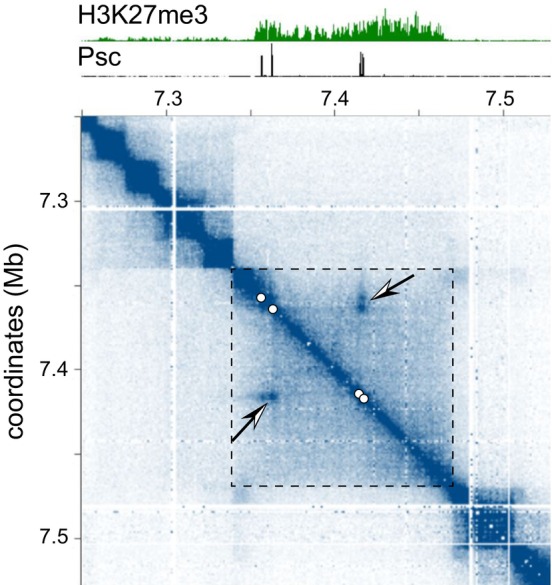
Chromatin contacts within the repressed *invected‐engrailed* locus. The heatmap representation of the chromatin contacts along a segment of *Drosophila* chromosome 2R (dark blue – more contacts, light blue – fewer contacts) gives an example of elevated contacts between Polycomb Response Elements (PREs) and the relation between H3K27me3 and Topologically Associated Domains (TADs) [80]. The profiles above the heatmap show ChIP‐seq signals for the PRC1 subunit Psc [37] and H3K27me3 [110]. The elevated ChIP‐seq signal for H3K27me3 delimits the repressed *invected‐engrailed* locus (also highlighted in the heatmap with dashed box edges). The four PREs of the locus (marked by the ChIP‐seq signal peaks for Psc and white circles on the heatmap) appear as dots (indicated with arrows) on the heatmap, which suggests their spatial proximity. Note that the right edge of the H3K27me3‐enriched *invected‐engrailed* locus corresponds to the TAD border, while the left edge of the locus does not.

Hi‐C may be used to investigate chromatin folding inside the loci repressed by the Polycomb system. When applied to *Drosophila* or human cells, Hi‐C indicates that the repressed loci correspond to broad domains (up to a million base pairs) with similar chromatin contact frequencies, which slowly decay with the distance until the edge of the domain [49, 75]. Such chromatin contact patterns are often referred to as topologically associated domains (TADs) [76]. TADs encompassing the repressed loci are often (but not always) coextensive with stretches of chromatin enriched in H3K27me3 (Fig. 3) [75, 77]. Although these TADs can, at times, be split by chromatin insulators [78]. Strikingly, the Hi‐C contact maps of *Drosophila* chromatin display spots of elevated contacts corresponding to some of the PREs within H3K27me3‐enriched TADs [77, 79, 80]. The contact spots suggest that PREs of some (but not all) repressed loci end up in closer spatial proximity compared to neighbouring stretches of chromatin located at comparable linear distances.

**Fig. 4:**
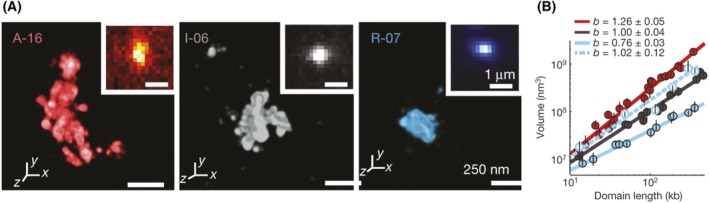
Optical reconstruction of local chromatin folding. (A) 3D‐STORM images of chromatin stretches (domains) in three distinct transcriptional states: ‘active’ (red), ‘inactive’ (grey), and ‘repressed’ (light blue). The insets show the corresponding conventional images The scale bars represent 1 μm (insets) and 250 nm (main panels). (B) log–log plot of the median chromatin volume as a function of domain length for active (red solid circles), inactive (black solid circles), and repressed (light blue solid circles) domains, as well as for repressed domains in Ph‐knockdown cells (light blue hollow circles). Error bars represent 95% confidence intervals derived from resampling (*n* ≈ 50 cells). The lines indicate power‐law fits, with the scaling exponent b shown in the legend. Figure reproduced from Ref. [66].

Super‐resolution microscopy combined with *in situ* hybridization of fluorescently labelled oligonucleotide probes provides alternative means to trace the 3D organization of chromatin inside the loci repressed by the Polycomb system. Applied to *Drosophila* embryos and cultured cells, such optical reconstructions indicate that the repressed loci are on average more crumpled compared to both transcriptionally active [66, 81, 82] and transcriptionally inactive, but not repressed, genes [66, 81]. The chromatin density inside the volume occupied by the repressed loci appears to increase with their length (Fig. 4) [66]. Moreover, the chromatin of the repressed loci seems to have a higher degree of chromatin intermixing [66, 81] and excludes the neighbouring transcriptionally active chromatin more than the chromatin of regular transcriptionally inactive genes [66]. Optical reconstruction of chromatin from murine *Hoxa* cluster repressed by the Polycomb system revealed essentially the same properties [83]. Although the loci repressed by the Polycomb system appear more compact, the corresponding volume occupied by a repressed locus is only about half of that for the similar‐sized transcriptionally inactive chromatin stretch.

Hi‐C assay requires pools of hundreds of thousands to millions of cells, so its estimate of the contact frequencies is an average over a large cell population. Optical reconstructions are inherently single‐cell techniques. But when averaged over many cells, these reconstructions show excellent correspondence with contact frequencies measured by Hi‐C [81, 82, 83]. However, they indicate that chromatin folding within loci repressed by the Polycomb system varies substantially between different cells. In particular, they argue that the chromatin of the repressed loci frequently assumes an unfolded state partially mixed with the neighbouring chromatin [81, 83]. Overall, the picture emerging from Hi‐C and super‐resolution microscopy is that of dynamic folding. In this view, the average more folded state of the chromatin within the repressed genes may arise from infrequent folding into an ultra‐compact conformation or in a lower propensity to assume a very unfolded state. Technical advances enabling high‐resolution live tracing of the chromatin motion inside loci repressed by the Polycomb system may allow us to discriminate between these possibilities.

What molecules and molecular forces lead to the local chromatin folding within the repressed loci? Here, the insights come from genetic, biochemical and computer simulation studies. The *in vitro* reconstituted PRC1 compacts nucleosomal arrays even when those contain no tethering elements (e.g. PREs) [84]. Consistent with this observation, multiple studies indicate that chromatin of the loci regulated by the system becomes less folded when one of the Polycomb group proteins is ablated by mutation [64, 66, 83, 85, 86]. The latter is customarily taken as proof that Polycomb group proteins are required for folding. However, the observed unfolding of a locus is often accompanied by an increase in transcriptional activity, which is known to unfold chromatin [66, 67]. Hence, unless this is considered, it is impossible to conclude whether the change in the chromatin structure precedes transcription or is the consequence of it. Two studies attempted to separate the effects of disrupting the Polycomb system and transcription. In one of them, the folding of *Drosophila* homeotic gene clusters was assayed in the early *Pc* or *Ph* loss‐of‐function embryos (mutants for one of the PRC1 subunits) when the clusters were still repressed by maternally supplied regulators independent of the Polycomb system [85]. In the other the chromatin folding of the *Hoxa* locus was reconstructed after simultaneous degradation of RING1B (PRC1 subunit) and EED (PRC2 subunit) in mouse embryonic stem cells, where this did not cause an increase of *Hoxa* transcription [83]. Both reported that chromatin unfolds without a detectable increase in transcription, which argues that the Polycomb group proteins are indeed the primary agents of the folding.

How could then the Polycomb group proteins fold chromatin? As discussed in the previous section, Polycomb group proteins Ph and Scm can form homo‐ and heteropolymers *in vitro* [62, 63]. It was hypothesized that the polymerization of Ph drives the chromatin folding within genes repressed by the Polycomb system [64]. The same may be suggested for Scm. It was also proposed that polymerization of Ph might mediate PRE clustering [77, 80]. Consistent with these hypotheses, reduced Ph concentration in the nucleus [66] led to reduced chromatin folding within genes regulated by the Polycomb system. However, the SAM domains of Ph and Scm are required for the repression [65, 87] and chromatin unfolding triggered by Ph removal or truncation was accompanied by increased transcription of the investigated genes. Therefore, from the above experiments alone, it is impossible to discriminate whether the changes in the chromatin structure occurred because of impaired Ph polymerization or due to transcription. Likewise, genetic evidence suggests that, in the cell, Ph has a limited ability to polymerize [65].

**Fig. 5:**
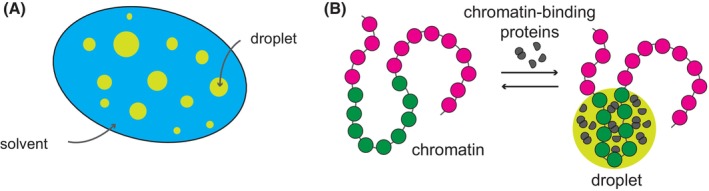
Liquid–liquid phase separation (LLPS) may aid chromatin folding. (A) Protein–protein or protein‐chromatin interactions lead to phase separation into liquid‐like droplets containing macromolecules of high concentration. LLPS is driven by the exchange of macromolecule/solvent interactions for macromolecule/macromolecule and solvent/solvent interactions when this becomes thermodynamically favourable. (B) Certain chromatin regions (green) may fold when bound by self‐interacting proteins (grey) when the latter phase separate and form droplets.

Liquid–liquid phase separation (LLPS) was hypothesized as another mechanism to aid local chromatin folding by the Polycomb system. LLPS is the process where macromolecules separate into a dense phase that resembles liquid droplets, which coexist with a dilute phase. LLPS is driven by the exchange of macromolecule/solvent interactions for macromolecule/macromolecule and solvent/solvent interactions when this becomes thermodynamically favourable (Fig. 5). The most thoroughly studied example of chromatin segregation by LLPS is the nucleolus, the membraneless nuclear organelle for ribosome subunit assembly [88]. LLPS provides an elegant way of increasing the local concentration of macromolecules which, unlike regular aggregation, does not sequester the mobility of the molecule inside the phase. The ability to undergo LLPS may be a universal property of proteins and nucleic acids under specific conditions, many of which may never be encountered in a cell [89]. Therefore, rigorous characterization of a macromolecule's behaviour *in vitro* and in the cell is required before one can confidently discriminate between LLPS and other mechanisms that may lead to a high local concentration of the molecule inside the nucleus. We point the reader to excellent reviews that discuss guidelines for experimental validation of LLPS [89, 90]. One of the mammalian PRC1 subunits (Cbx2) was reported to phase separate and drive phase separation of associated PRC1 and nucleosomal arrays *in vitro* [91, 92, 93]. However, the current evidence falls short of recommended guidelines [89, 90]. The reported LLPS‐like behaviour requires an intact intrinsically disordered region (IDR) of Cbx2, and mutations of the corresponding residues within IDR lead to homeotic transformations in mutant mice [94]. It remains to be tested whether Cbx2 undergoes LLPS *in vivo*, and it is not immediately clear how the presumed high local concentration of phase‐separated PRC1 translates into increased local chromatin folding. Further complicating the issue, of the closely related mammalian orthologues of *Drosophila* Polycomb protein (Cbx2, Cbx4, Cbx6 Cbx7 and Cbx8), only Cbx2 appears to phase separate *in vitro* by itself [92, 95] and Cbx8 as part of the PRC1 complex and in the presence of chromatin [96]. This implies that most paralogues have lost the ability for LLPS or that this ability evolved after the paralogues diverged from their common ancestor (i.e. after the Polycomb system evolved to epigenetically repress developmental genes). Whether *Drosophila* Polycomb protein can phase separate *in vitro* has not been tested.

**Fig. 6:**
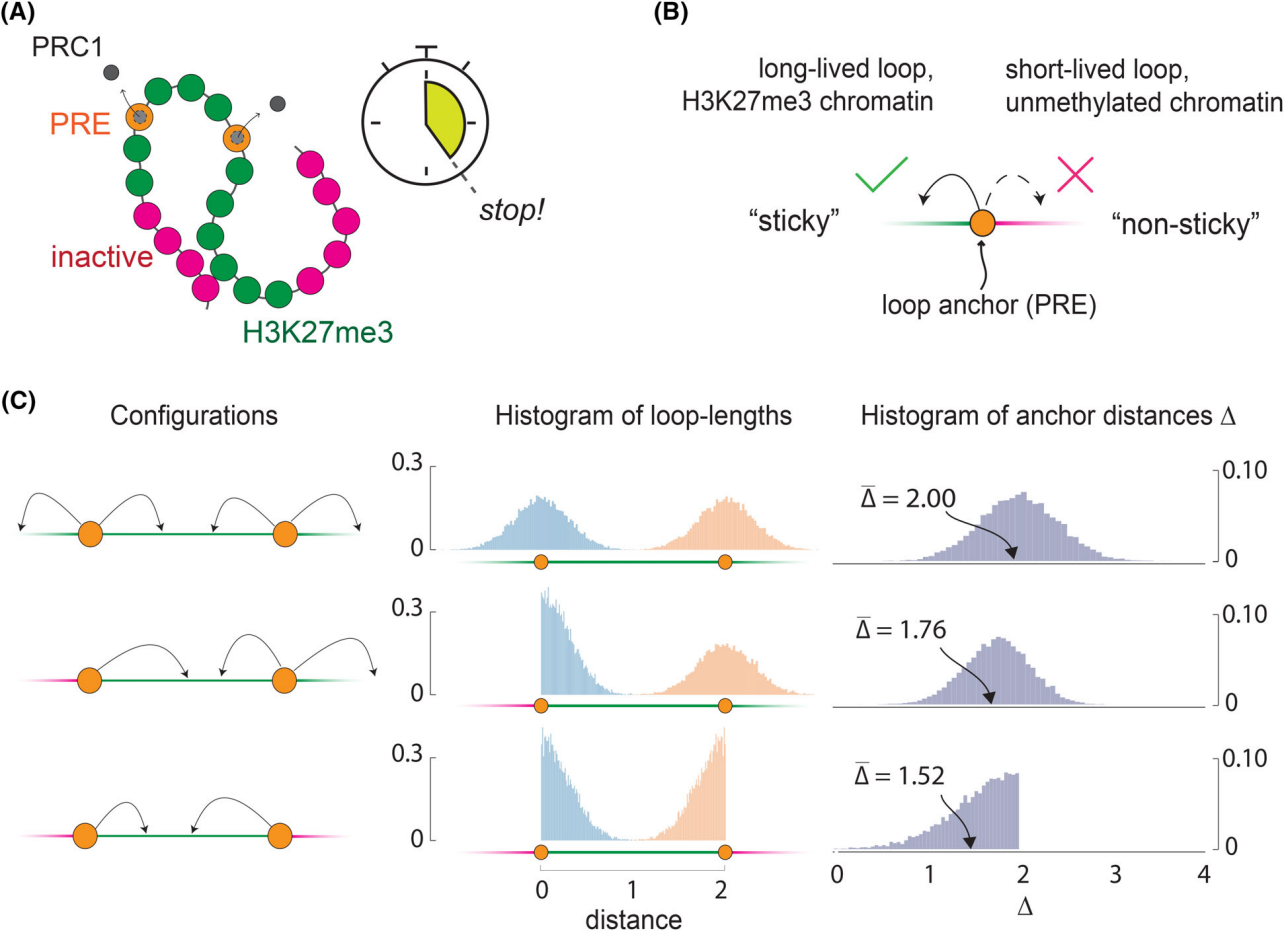
Chromatin folding by PRC1‐H3K27me3 interactions leads to spatial clustering of Polycomb Response Elements (PREs). (A) Schematic of the pseudo‐nonequilibrium block copolymer model of Lizana et al. [97]. PRE monomers (orange) attract PRC1 complexes (grey), and stochastic interactions between PRC1 and H3K27me3 fold the methylated chromatin. The simulation stops after a short time, comparable to the PRC1 residence time at PREs. (B) Simple looping model. It considers anchor points (orange circles) on a line that may touch surrounding line segments and form loops. Depending on the segment type, “sticky” (green) or “non‐sticky” (red), the loops will be long or short‐lived. The model postulates that only long‐lived loops are stable enough to be detected. (C) Three basic configurations of anchor points and chromatin types. Arrows indicate the directions in which the anchor points may form long‐lived loops. Shown to the right are the corresponding histograms of the simulated loop lengths and anchor distances. The average anchor distance ($\bar{\Delta }$) depends on the relative arrangement of anchor points and the “sticky” chromatin segments. Panel C is reproduced from Ref. [97].

Lastly, it appears possible that the chromatin of genes repressed by the Polycomb system is folded just by stochastic interactions of PRE‐bound PRC1 with histone H3 trimethylated at lysine 27. A recent computational study from our laboratories used a biochemically grounded Monte Carlo – Molecular Dynamics simulation framework to model the chromatin folding within *Drosophila* PRE‐equipped genes [97]. Unlike traditional block copolymer models discussed earlier, this pseudo‐nonequilibrium model introduced a ‘new’ PRC1‐H3K27me3 interaction to a chromatin fibre that has been in equilibrium, proceeded with molecular dynamics simulations but stopped them after a short time comparable to that for PRC1 residence at PREs (Fig. 6A). By scanning a range of binding constants for PRC1 – PRE and PRC1‐H3K27me3 interactions, the study demonstrates that stochastic interactions of PRE‐tethered PRC1 with H3K27me3 are sufficient to fold the methylated chromatin, if PREs are occupied by PRC1 most of the time. In line with the results of optical reconstructions [81, 83], simulations indicate that the extent of folding varies substantially between individual chromatin fibres. The model makes a testable prediction. In cells where PRC1 is truncated so it can no longer interact with H3K27me3 or where H3K27 methylation is removed by PRC2 mutation, PRC1 will continue to bind PREs, but the chromatin folding will be lost. Such genetic and imaging experiments have not yet been performed. However, genomic studies in PRC2 knock‐out cells do show that, in the absence of H3K27me3, PRC1 continues to bind PREs while its interaction with the surrounding chromatin becomes less stable [37].

Remarkably, chromatin folding by PRC1‐H3K27me3 interactions leads to spatial clustering of PREs, noted in Hi‐C experiments [77, 79, 80], without a need for specific protein–protein interactions. The clustering appears to have a geometric/probabilistic explanation, which we briefly discuss below and refer the interested reader to the original study for details [97]. Let us consider PRC1‐bound PREs as points on a line (Fig. 6B) that may touch surrounding line segments and form loops. Depending on the segment type, which could be ‘sticky’ (e.g. H3K27 trimethylated chromatin) or “non‐sticky” (e.g. unmethylated chromatin), the loop will be long‐lived or short‐lived. If we consider the behaviour of two PRC1‐bound PREs, they can be found in three basic configurations (Fig. 6C). In the first configuration, the ‘sticky’ segments surround both PREs. As a result, those may form stable loops to the left and right with the same probability (the probability to touch a specific segment point declines with its distance from a PRE, and the exact dependence is not essential for the argument). As illustrated by Fig. 6C, this configuration yields a symmetric distribution of loop lengths and absolute distances (Δ) between the two anchor points. As a result, the average distance between the PREs ($\bar{\Delta }$) equals 2, which is identical to their linear separation. In the second configuration, the leftmost segment is ‘non‐sticky’, while the other segments remain the same as in the first configuration. Since, in this arrangement, no long‐lived loops form towards the leftmost segment, the corresponding loop length histogram is asymmetric, and the average distance between the PREs shortens to $\bar{\Delta }$ = 1.76. Compared to the first configuration, PREs become statistically closer. Lastly, we consider the most extreme configuration where interactions with only one segment between the two PREs can yield stable loops. In this arrangement, the average distance between PREs shortens even more to $\bar{\Delta }$ = 1.52.

To summarize, Polycomb group proteins appear to increase local chromatin folding. However, the resulting chromatin compaction is modest (no more than 2‐fold on average) and dynamic. The chromatin of genes repressed by the Polycomb system remains free to explore unfolded states and periodically mix with the chromatin of neighbouring genes. This folding behaviour is incompatible with the model where Polycomb group proteins repress transcription by compacting the chromatin to a point when it becomes inaccessible to transcriptional activators and transcriptional machinery. In line with this view, attempts to correlate the repression of a locus by the Polycomb system to the loss of DNA accessibility yielded conflicting results. Thus, transgenic reporters repressed by the Polycomb system are harder to methylate by exogenously expressed Dam DNA methylase [98] or excise with FLP recombinase [99]. Yet, DNA of homeotic genes is equally accessible to restriction nucleases in transcriptionally active and repressed states [100], and although 5′ ends of the genes occupied by PRC1 and PRC2 in mouse embryonic stem cells exhibit reduced DNA accessibility compared to those that do not bind the complexes, the accessibility does not increase after PRC1 or PRC2 are ablated [101]. Likewise, PREs of genes repressed by the Polycomb system are DNase I hypersensitive [36] and display elevated nucleosome exchange [33, 102, 103]. The repression does not prevent the binding of TATA binding protein (TBP), RNA polymerase II, and the heat‐shock factor to a reporter gene driven by the hsp26 promoter [104], and does not hinder insulator proteins from binding to insulator elements [78].

## Implications for epigenetic repression

**Fig. 7:**
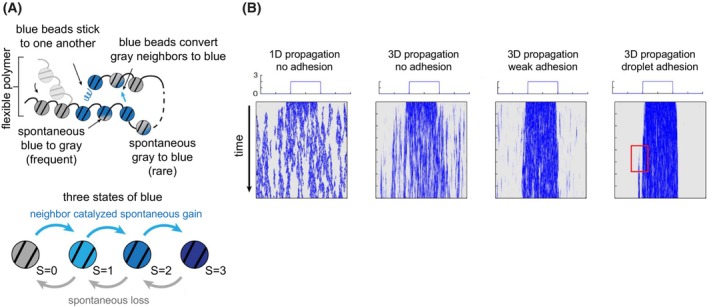
Polymer modelling argues that chromatin folding by the Polycomb system helps to propagate the epigenetic memory. (A) Schematic illustrating key features of the model of Murphy and Boettiger [83]. (B) Kymographs from representative simulations. The X‐axis reports monomer position (genomic coordinate), and the Y‐axis reports simulation time (arbitrary units). The starting state of the simulation is shown above the kymograph, along with the degree of adhesion between blue monomers. Note the propagation of epigenetic memory (e.g. a stretch of chromatin enriched in methylated H3K27) without stable compaction on the second kymograph from the right. The red box in the rightmost kymograph highlights a monomer outside the repressed locus that acquired methylated H3K27 by accident. Figure reproduced from Ref. [83].

If the chromatin folding by the Polycomb system does not hinder the access of transcriptional activators and transcriptional machinery to chromatin, is it critical for epigenetic repression, and if so, how does the folding promote it? Using computational modelling, two recent studies argue that chromatin folding by the Polycomb system is critical to propagating epigenetic memory [83, 105]. The model of Murphy and Boettiger represents chromatin as a flexible polymer where each segment (monomer) can switch epigenetic states (acquire methyl groups on H3K27) dynamically over time—from unmethylated to mono‐, di‐, or trimethylation (Fig. 7). The switching happens at some constant rate but only if other methylated monomers are brought into spatial proximity. The loss of methylation (demethylation) occurs randomly. Furthermore, each methylated state is associated with an adhesion parameter that represents the interaction between the Polycomb group proteins bound to methylated histones, presumably PRC1 and PRC2. This parameter determines how tightly methylated monomers stick together if meeting in 3D space. From simulations, the model predicts a delicate balance between chromatin folding and flexibility. If the adhesion is too weak, a locus repressed by the Polycomb system loses H3K27 methylation because the methylation spreads only in 1D, which is slow, and eventually gets lost due to random demethylation. But if the adhesion is sufficiently high, methylated monomers are on average closer to each other, leading to more efficient methylation spreading and tighter folding in a self‐reinforcing feedback loop. This process results in a long‐lived epigenetic state with bi‐stable characteristics. It can restore erroneous loss of epigenetic states for individual monomers and recover from cell division that reduces the density of methylated monomers by 50%. However, having a too high adhesion parameter comes with a cost. If a monomer outside the repressed locus acquires methylated H3K27 by accident, it fuses with the other methylated monomers and its methylated state is perpetuated indefinitely. To summarize, the model predicts that the chromatin folding by the Polycomb system must remain balanced. It should be efficient enough to maintain a long‐lived methylation state of the repressed locus, but not too strong so it sustains erroneous methylation of the flanking chromatin [83]. Qualitatively similar conclusions follow from a more general model of Owen and colleagues [105]. In this case, the authors emphasize that locus‐specific, yet epigenetically stable repression requires that the marking enzyme (e.g. PRC2) is limited relative to its substrate (nucleosomes).

While coherent, neither of the above models considers that PRC2 (the marking enzyme) and PRC1, which enables chromatin folding, are tethered to specific genes by DNA elements (PREs in *Drosophila* or CpG‐islands and PTEs in mammals). Experimentally induced deletion of tethering elements leads to the rapid loss of H3K27 methylation and de‐repression [106, 107]. Thus, models that explain the propagation of epigenetic memory through self‐attraction and 3D spreading of H3K27 methylation are considerable simplifications. Perhaps, they imply tethering as part of molecular processes that enable the self‐attraction and/or methylation spreading. Conversely, the probabilistic model grounded in the enzymatic properties of PRC2 in the solution that accounts for H3K27 methylation by PRE‐tethered complexes can forecast genomic H3K27 methylation with the accuracy of current experimental techniques [108]. This model incorporates chromatin folding around PREs implicitly when it converts the degree of PRC2 tethering at PRE‐equipped genes into higher methylation probability.

To summarize, the local chromatin folding by the Polycomb group proteins is likely required for extensive trimethylation of H3K27 around tethering points (i.e. PREs) and the sufficient speed with which the H3K27me3 is regained after chromatin replication. Together with the allosteric stimulation by the pre‐existing H3K27me3 [109], the folding makes PRC2 better at methylating H3K27 at sites where it is needed the most (i.e. developmental genes equipped with tethering elements) as opposed to indiscriminate methylation of the entire transcriptionally inactive genome [108, 110]. It is tempting to speculate that the impact of local chromatin folding is not limited to the propagation of the epigenetic memory record. Exploratory computational modelling from our laboratories suggests that stochastic interactions of PRE‐bound PRC1 with the surrounding H3K27me3 slow down the transitions of the chromatin fibre from one folding conformation to another. If transcription initiation happens only in certain infrequent folding conformations, reduced chromatin fibre dynamics would inhibit the process. In line with this view, a recent live‐cell transcription imaging study in mouse embryonic stem cells indicates that genes repressed by the Polycomb system alternate between transcriptionally active and inactive states [111]. While the system does not constrain the transcription in the active state, it makes this state extremely infrequent [111]. More work is needed to extend these observations and test their possible connection to local chromatin folding.

*Trans*‐interactions between genes repressed by the Polycomb system can boost the repression. As discussed earlier, PRE‐containing transgenic reporters exhibit ‘paring‐sensitive silencing’, a phenomenon where *Drosophila* homozygous for the transgenic allele express the reporter less than those heterozygous for the allele [40, 41]. Even though, in the former case, the files carry two copies of the transgene as opposed to one present in heterozygotes. Pairing‐sensitive silencing requires that the two transgenes are in spatial proximity, which, in the cases above, arises from somatic pairing of homologous chromosomes. The pairing‐sensitive silencing can happen even between two transgenes inserted in different chromosomes. In those cases, the transgenes must contain a chromatin insulator element to promote *trans*‐interactions [42]. Overall, the spectacular and well‐documented cases of pairing‐sensitive silencing involve the side force helping to bring the repressed genes in spatial proximity. To what extent the long‐range chromatin interactions mediated by the Polycomb group proteins contribute to the known cases of pairing‐sensitive silencing is an open question. Likewise, it remains to be seen whether long‐range interactions are important to repress genes at their native genomic locations. We are unaware of cases where a deletion or a translocation of one copy of a gene regulated by the Polycomb system led to the de‐repression of the unaffected allele. Undoubtedly, more work is needed to gain a mechanistic understanding of how the long‐range interactions between the repressed genes affect the transcription.

## Concluding remarks

Recent observations using Hi‐C, super‐resolution microscopy, and polymer modelling leave little doubt that Polycomb group proteins increase the local chromatin folding. Stochastic interactions of tethered PRC1 with H3K27me3 or interactions between PRC1 complexes emerged as the prime candidates that enable the process. Remarkably, the resulting chromatin compaction is rather modest (no more than 2‐fold on average) and dynamic. This behaviour questions traditional models where Polycomb group proteins repress transcription by restricting access for transcriptional activators and transcriptional machinery. How such dynamic chromatin folding enhances the epigenetic repression or whether the folding is just an ineffectual byproduct of molecular interactions within the Polycomb system are important open questions for future research. The emerging live imaging methods and computational modelling are the approaches most likely to bridge these gaps.

Likewise, genes repressed by the Polycomb system tend to be closer to each other in the nuclear space than expected by chance. However, this effect is even subtler than local chromatin folding. Whether such spatial proximity involves molecular interactions between the Polycomb group proteins bound to the ‘paired’ genes remains to be seen. In contrast to local chromatin folding, many lines of evidence indicate that the proximity between distinct genes promotes repression. Yet, the reported effects have been limited to transgenes and required an additional force to bring the repressed genes in spatial proximity. Testing the relative contribution of long‐range interactions to the repression of genes at their endogenous locations is a challenging problem to solve in the coming years.

## Conflict of interest

The authors declare that they have no competing interests.

## Author contributions

Conceptualization: LL and YBS. Writing original draft: LL and YBS. Review and editing: LL and YBS.

## References

[1] Deevy O & Bracken AP (2019) PRC2 functions in development and congenital disorders. Development 146, dev181354. 31575610 10.1242/dev.181354 PMC6803372

[2] Piunti A & Shilatifard A (2021) The roles of Polycomb repressive complexes in mammalian development and cancer. Nat Rev Mol Cell Biol 22, 326–345. 33723438 10.1038/s41580-021-00341-1

[3] Jürgens G (1985) A group of genes controlling the spatial expression of the bithorax complex in drosophila. Nature 316, 153–155.

[4] Lewis EB (1978) A gene complex controlling segmentation in drosophila. Nature 276, 565–570. 103000 10.1038/276565a0

[5] Struhl G & Brower D (1982) Early role of the *esc* ^+^ gene product in the determination of segments in *drosophila* . Cell 31, 285–292. 7159925 10.1016/0092-8674(82)90428-7

[6] Czermin B , Melfi R , McCabe D , Seitz V , Imhof A & Pirrotta V (2002) Drosophila enhancer of Zeste/ESC complexes have a histone H_3_ methyltransferase activity that marks chromosomal Polycomb sites. Cell 111, 185–196. 12408863 10.1016/s0092-8674(02)00975-3

[7] Kuzmichev A , Nishioka K , Erdjument‐Bromage H , Tempst P & Reinberg D (2002) Histone methyltransferase activity associated with a human multiprotein complex containing the enhancer of Zeste protein. Genes Dev 16, 2893–2905. 12435631 10.1101/gad.1035902 PMC187479

[8] Levine SS , Weiss A , Erdjument‐Bromage H , Shao Z , Tempst P & Kingston RE (2002) The core of the polycomb repressive complex is compositionally and functionally conserved in flies and humans. Mol Cell Biol 22, 6070–6078. 12167701 10.1128/MCB.22.17.6070-6078.2002 PMC134016

[9] Shao Z , Raible F , Mollaaghababa R , Guyon JR , Wu CT , Bender W & Kingston RE (1999) Stabilization of chromatin structure by PRC1, a Polycomb complex. Cell 98, 37–46. 10412979 10.1016/S0092-8674(00)80604-2

[10] Kim JJ & Kingston RE (2022) Context‐specific Polycomb mechanisms in development. Nat Rev Genet 23, 680–695. 35681061 10.1038/s41576-022-00499-0 PMC9933872

[11] Lizana L & Schwartz YB (2024) The scales, mechanisms, and dynamics of the genome architecture. Sci Adv 10, eadm8167. 38598632 10.1126/sciadv.adm8167 PMC11006219

[12] Muller J , Hart CM , Francis NJ , Vargas ML , Sengupta A , Wild B , Miller EL , O'Connor MB , Kingston RE & Simon JA (2002) Histone methyltransferase activity of a drosophila Polycomb group repressor complex. Cell 111, 197–208. 12408864 10.1016/s0092-8674(02)00976-5

[13] Pengelly AR , Copur O , Jackle H , Herzig A & Muller J (2013) A histone mutant reproduces the phenotype caused by loss of histone‐modifying factor Polycomb. Science 339, 698–699. 23393264 10.1126/science.1231382

[14] Sankar A , Mohammad F , Sundaramurthy AK , Wang H , Lerdrup M , Tatar T & Helin K (2022) Histone editing elucidates the functional roles of H3K27 methylation and acetylation in mammals. Nat Genet 54, 754–760. 35668298 10.1038/s41588-022-01091-2

[15] Gao Z , Zhang J , Bonasio R , Strino F , Sawai A , Parisi F , Kluger Y & Reinberg D (2012) PCGF homologs, CBX proteins, and RYBP define functionally distinct PRC1 family complexes. Mol Cell 45, 344–356. 22325352 10.1016/j.molcel.2012.01.002 PMC3293217

[16] Hauri S , Comoglio F , Seimiya M , Gerstung M , Glatter T , Hansen K , Aebersold R , Paro R , Gstaiger M & Beisel C (2016) A high‐density map for navigating the human Polycomb Complexome. Cell Rep 17, 583–595. 27705803 10.1016/j.celrep.2016.08.096

[17] Kang H , Cabrera JR , Zee BM , Kang HA , Jobe JM , Hegarty MB , Barry AE , Glotov A , Schwartz YB & Kuroda MI (2022) Variant Polycomb complexes in drosophila consistent with ancient functional diversity. Sci Adv 8, eadd0103. 36070387 10.1126/sciadv.add0103 PMC9451159

[18] Kloet SL , Makowski MM , Baymaz HI , van Voorthuijsen L , Karemaker ID , Santanach A , Jansen P , Di Croce L & Vermeulen M (2016) The dynamic interactome and genomic targets of Polycomb complexes during stem‐cell differentiation. Nat Struct Mol Biol 23, 682–690. 27294783 10.1038/nsmb.3248 PMC4939079

[19] Cao R , Tsukada Y & Zhang Y (2005) Role of Bmi‐1 and Ring1A in H2A ubiquitylation and Hox gene silencing. Mol Cell 20, 845–854. 16359901 10.1016/j.molcel.2005.12.002

[20] Taherbhoy AM , Huang OW & Cochran AG (2015) BMI1‐RING1B is an autoinhibited RING E3 ubiquitin ligase. Nat Commun 6, 7621. 26151332 10.1038/ncomms8621

[21] Akasaka T , Lohuizen M , Lugt N , Mizutani‐Koseki Y , Kanno M , Taniguchi M , Vidal M , Alkema M , Berns A & Koseki H (2001) Mice doubly deficient for the Polycomb group genes Mel18 and Bmi1 reveal synergy and requirement for maintenance but not initiation of Hox gene expression. Development 128, 1587–1597. 11290297 10.1242/dev.128.9.1587

[22] Bel S , Core N , Djabali M , Kieboom K , Van der Lugt N , Alkema MJ & Van Lohuizen M (1998) Genetic interactions and dosage effects of Polycomb group genes in mice. Development 125, 3543–3551. 9716520 10.1242/dev.125.18.3543

[23] Isono K , Fujimura Y , Shinga J , Yamaki M , Takihara Y , Murahashi Y , Takada Y , Mizutani‐Koseki Y & Koseki H (2005) Mammalian polyhomeotic homologues Phc2 and Phc1 act in synergy to mediate polycomb repression of Hox genes. Mol Cell Biol 25, 6694–6706. 16024804 10.1128/MCB.25.15.6694-6706.2005 PMC1190356

[24] Oktaba K , Gutierrez L , Gagneur J , Girardot C , Sengupta AK , Furlong EE & Muller J (2008) Dynamic regulation by polycomb group protein complexes controls pattern formation and the cell cycle in drosophila. Dev Cell 15, 877–889. 18993116 10.1016/j.devcel.2008.10.005

[25] van der Lugt NM , Domen J , Linders K , van Roon M , Robanus‐Maandag E , te Riele H , van der Valk M , Deschamps J , Sofroniew M & van Lohuizen M (1994) Posterior transformation, neurological abnormalities, and severe hematopoietic defects in mice with a targeted deletion of the bmi‐1 proto‐oncogene. Genes Dev 8, 757–769. 7926765 10.1101/gad.8.7.757

[26] Blackledge NP , Fursova NA , Kelley JR , Huseyin MK , Feldmann A & Klose RJ (2020) PRC1 catalytic activity is central to Polycomb system function. Mol Cell 77, 857–874. 31883950 10.1016/j.molcel.2019.12.001 PMC7033600

[27] Bonnet J , Boichenko I , Kalb R , Le Jeune M , Maltseva S , Pieropan M , Finkl K , Fierz B & Muller J (2022) PR‐DUB preserves Polycomb repression by preventing excessive accumulation of H2Aub1, an antagonist of chromatin compaction. Genes Dev 36, 1046–1061. 36357125 10.1101/gad.350014.122 PMC9744231

[28] Illingworth RS , Moffat M , Mann AR , Read D , Hunter CJ , Pradeepa MM , Adams IR & Bickmore WA (2015) The E3 ubiquitin ligase activity of RING1B is not essential for early mouse development. Genes Dev 29, 1897–1902. 26385961 10.1101/gad.268151.115 PMC4579347

[29] Pengelly AR , Kalb R , Finkl K & Muller J (2015) Transcriptional repression by PRC1 in the absence of H2A monoubiquitylation. Genes Dev 29, 1487–1492. 26178786 10.1101/gad.265439.115 PMC4526733

[30] Scelfo A , Fernandez‐Perez D , Tamburri S , Zanotti M , Lavarone E , Soldi M , Bonaldi T , Ferrari KJ & Pasini D (2019) Functional landscape of PCGF proteins reveals both RING1A/B‐dependent‐and RING1A/B‐independent‐specific activities. Mol Cell 74, 1037–1052. 31029542 10.1016/j.molcel.2019.04.002 PMC6561742

[31] Tamburri S , Lavarone E , Fernandez‐Perez D , Conway E , Zanotti M , Manganaro D & Pasini D (2020) Histone H2AK119 mono‐ubiquitination is essential for Polycomb‐mediated transcriptional repression. Mol Cell 77, 840–856. 31883952 10.1016/j.molcel.2019.11.021 PMC7033561

[32] Schwartz YB (2017) Cooperative recruitment of Polycomb complexes by Polycomb response elements. Polycomb Group Proteins 111–129. 10.1016/b978-0-12-809737-3.00006-4

[33] Barrasa JI , Kahn TG , Lundkvist MJ & Schwartz YB (2023) DNA elements tether canonical Polycomb repressive complex 1 to human genes. Nucleic Acids Res 51, 11613–11633. 37855680 10.1093/nar/gkad889 PMC10681801

[34] Wang L , Brown JL , Cao R , Zhang Y , Kassis JA & Jones RS (2004) Hierarchical recruitment of polycomb group silencing complexes. Mol Cell 14, 637–646. 15175158 10.1016/j.molcel.2004.05.009

[35] Schwartz YB , Kahn TG , Nix DA , Li XY , Bourgon R , Biggin M & Pirrotta V (2006) Genome‐wide analysis of Polycomb targets in *Drosophila melanogaster* . Nat Genet 38, 700–705. 16732288 10.1038/ng1817

[36] Kharchenko PV , Alekseyenko AA , Schwartz YB , Minoda A , Riddle NC , Ernst J , Sabo PJ , Larschan E , Gorchakov AA , Gu T *et al*. (2011) Comprehensive analysis of the chromatin landscape in *Drosophila melanogaster* . Nature 471, 480–485. 21179089 10.1038/nature09725 PMC3109908

[37] Kahn TG , Dorafshan E , Schultheis D , Zare A , Stenberg P , Reim I , Pirrotta V & Schwartz YB (2016) Interdependence of PRC1 and PRC2 for recruitment to Polycomb response elements. Nucleic Acids Res 44, 10132–10149. 27557709 10.1093/nar/gkw701 PMC5137424

[38] Alkema MJ , Bronk M , Verhoeven E , Otte A , van 't Veer LJ , Berns A & van Lohuizen M (1997) Identification of Bmi1‐interacting proteins as constituents of a multimeric mammalian polycomb complex. Genes Dev 11, 226–240. 9009205 10.1101/gad.11.2.226

[39] Buchenau P , Hodgson J , Strutt H & Arndt‐Jovin DJ (1998) The distribution of polycomb‐group proteins during cell division and development in drosophila embryos: impact on models for silencing. J Cell Biol 141, 469–481. 9548724 10.1083/jcb.141.2.469 PMC2148446

[40] Kassis JA (1994) Unusual properties of regulatory DNA from the drosophila engrailed gene: three “pairing‐sensitive” sites within a 1.6‐kb region. Genetics 136, 1025–1038. 8005412 10.1093/genetics/136.3.1025 PMC1205860

[41] Kassis JA , VanSickle EP & Sensabaugh SM (1991) A fragment of engrailed regulatory DNA can mediate transvection of the white gene in drosophila. Genetics 128, 751–761. 1655566 10.1093/genetics/128.4.751 PMC1204549

[42] Sigrist CJ & Pirrotta V (1997) Chromatin insulator elements block the silencing of a target gene by the drosophila polycomb response element (PRE) but allow trans interactions between PREs on different chromosomes. Genetics 147, 209–221. 9286681 10.1093/genetics/147.1.209 PMC1208105

[43] Sati S & Cavalli G (2017) Chromosome conformation capture technologies and their impact in understanding genome function. Chromosoma 126, 33–44. 27130552 10.1007/s00412-016-0593-6

[44] Bantignies F , Roure V , Comet I , Leblanc B , Schuettengruber B , Bonnet J , Tixier V , Mas A & Cavalli G (2011) Polycomb‐dependent regulatory contacts between distant Hox loci in Drosophila. Cell 144, 214–226. 21241892 10.1016/j.cell.2010.12.026

[45] Isono K , Endo TA , Ku M , Yamada D , Suzuki R , Sharif J , Ishikura T , Toyoda T , Bernstein BE & Koseki H (2013) SAM domain polymerization links subnuclear clustering of PRC1 to gene silencing. Dev Cell 26, 565–577. 24091011 10.1016/j.devcel.2013.08.016

[46] Kraft K , Yost KE , Murphy SE , Magg A , Long Y , Corces MR , Granja JM , Wittler L , Mundlos S , Cech TR *et al*. (2022) Polycomb‐mediated genome architecture enables long‐range spreading of H3K27 methylation. Proc Natl Acad Sci USA 119, e2201883119. 35617427 10.1073/pnas.2201883119 PMC9295753

[47] Rhodes JDP , Feldmann A , Hernandez‐Rodriguez B , Diaz N , Brown JM , Fursova NA , Blackledge NP , Prathapan P , Dobrinic P , Huseyin MK *et al*. (2020) Cohesin disrupts Polycomb‐dependent chromosome interactions in embryonic stem cells. Cell Rep 30, 820–835. 31968256 10.1016/j.celrep.2019.12.057 PMC6988126

[48] Schoenfelder S , Sugar R , Dimond A , Javierre BM , Armstrong H , Mifsud B , Dimitrova E , Matheson L , Tavares‐Cadete F , Furlan‐Magaril M *et al*. (2015) Polycomb repressive complex PRC1 spatially constrains the mouse embryonic stem cell genome. Nat Genet 47, 1179–1186. 26323060 10.1038/ng.3393 PMC4847639

[49] Sexton T , Yaffe E , Kenigsberg E , Bantignies F , Leblanc B , Hoichman M , Parrinello H , Tanay A & Cavalli G (2012) Three‐dimensional folding and functional organization principles of the drosophila genome. Cell 148, 458–472. 22265598 10.1016/j.cell.2012.01.010

[50] Tolhuis B , Blom M , Kerkhoven RM , Pagie L , Teunissen H , Nieuwland M , Simonis M , de Laat W , van Lohuizen M & van Steensel B (2011) Interactions among Polycomb domains are guided by chromosome architecture. PLoS Genet 7, e1001343. 21455484 10.1371/journal.pgen.1001343 PMC3063757

[51] Gurgo J , Walter JC , Fiche JB , Houbron C , Schaeffer M , Cavalli G , Bantignies F & Nollmann M (2024) Multiplexed chromatin imaging reveals predominantly pairwise long‐range coordination between drosophila Polycomb genes. Cell Rep 43, 114167. 38691452 10.1016/j.celrep.2024.114167

[52] Cheutin T & Cavalli G (2012) Progressive polycomb assembly on H3K27me3 compartments generates polycomb bodies with developmentally regulated motion. PLoS Genet 8, e1002465. 22275876 10.1371/journal.pgen.1002465 PMC3262012

[53] Belaghzal H , Borrman T , Stephens AD , Lafontaine DL , Venev SV , Weng Z , Marko JF & Dekker J (2021) Liquid chromatin hi‐C characterizes compartment‐dependent chromatin interaction dynamics. Nat Genet 53, 367–378. 33574602 10.1038/s41588-021-00784-4 PMC7946813

[54] Chiariello AM , Bianco S , Esposito A , Fiorillo L , Conte M , Irani E , Musella F , Abraham A , Prisco A & Nicodemi M (2022) Physical mechanisms of chromatin spatial organization. FEBS J 289, 1180–1190. 33583147 10.1111/febs.15762

[55] Fudenberg G , Imakaev M , Lu C , Goloborodko A , Abdennur N & Mirny LA (2016) Formation of chromosomal domains by loop extrusion. Cell Rep 15, 2038–2049. 27210764 10.1016/j.celrep.2016.04.085 PMC4889513

[56] Sanborn AL , Rao SS , Huang SC , Durand NC , Huntley MH , Jewett AI , Bochkov ID , Chinnappan D , Cutkosky A , Li J *et al*. (2015) Chromatin extrusion explains key features of loop and domain formation in wild‐type and engineered genomes. Proc Natl Acad Sci USA 112, E6456–E6465. 26499245 10.1073/pnas.1518552112 PMC4664323

[57] Jost D , Carrivain P , Cavalli G & Vaillant C (2014) Modeling epigenome folding: formation and dynamics of topologically associated chromatin domains. Nucleic Acids Res 42, 9553–9561. 25092923 10.1093/nar/gku698 PMC4150797

[58] Mirny LA (2011) The fractal globule as a model of chromatin architecture in the cell. Chromosome Res 19, 37–51. 21274616 10.1007/s10577-010-9177-0 PMC3040307

[59] Lieberman‐Aiden E , van Berkum NL , Williams L , Imakaev M , Ragoczy T , Telling A , Amit I , Lajoie BR , Sabo PJ , Dorschner MO *et al*. (2009) Comprehensive mapping of long‐range interactions reveals folding principles of the human genome. Science 326, 289–293. 19815776 10.1126/science.1181369 PMC2858594

[60] Kang H , McElroy KA , Jung YL , Alekseyenko AA , Zee BM , Park PJ & Kuroda MI (2015) Sex comb on midleg (Scm) is a functional link between PcG‐repressive complexes in drosophila. Genes Dev 29, 1136–1150. 26063573 10.1101/gad.260562.115 PMC4470282

[61] Maezawa S , Hasegawa K , Yukawa M , Kubo N , Sakashita A , Alavattam KG , Sin HS , Kartashov AV , Sasaki H , Barski A *et al*. (2018) Polycomb protein SCML2 facilitates H3K27me3 to establish bivalent domains in the male germline. Proc Natl Acad Sci USA 115, 4957–4962. 29686098 10.1073/pnas.1804512115 PMC5949012

[62] Kim CA , Gingery M , Pilpa RM & Bowie JU (2002) The SAM domain of polyhomeotic forms a helical polymer. Nat Struct Biol 9, 453–457. 11992127 10.1038/nsb802

[63] Kim CA , Sawaya MR , Cascio D , Kim W & Bowie JU (2005) Structural organization of a sex‐comb‐on‐midleg/polyhomeotic copolymer. J Biol Chem 280, 27769–27775. 15905166 10.1074/jbc.M503055200

[64] Wani AH , Boettiger AN , Schorderet P , Ergun A , Munger C , Sadreyev RI , Zhuang X , Kingston RE & Francis NJ (2016) Chromatin topology is coupled to Polycomb group protein subnuclear organization. Nat Commun 7, 10291. 26759081 10.1038/ncomms10291 PMC4735512

[65] Gambetta MC & Muller J (2014) O‐GlcNAcylation prevents aggregation of the Polycomb group repressor polyhomeotic. Dev Cell 31, 629–639. 25468754 10.1016/j.devcel.2014.10.020

[66] Boettiger AN , Bintu B , Moffitt JR , Wang S , Beliveau BJ , Fudenberg G , Imakaev M , Mirny LA , Wu CT & Zhuang X (2016) Super‐resolution imaging reveals distinct chromatin folding for different epigenetic states. Nature 529, 418–422. 26760202 10.1038/nature16496 PMC4905822

[67] Simon JA , Sutton CA & Lis JT (1985) Localization and expression of transformed DNA sequences within heat shock puffs of *Drosophila melanogaster* . Chromosoma 93, 26–30. 3933923 10.1007/BF01259442

[68] Gambetta MC , Oktaba K & Muller J (2009) Essential role of the glycosyltransferase sxc/Ogt in polycomb repression. Science 325, 93–96. 19478141 10.1126/science.1169727

[69] Clow PA , Du M , Jillette N , Taghbalout A , Zhu JJ & Cheng AW (2022) CRISPR‐mediated multiplexed live cell imaging of nonrepetitive genomic loci with one guide RNA per locus. Nat Commun 13, 1871. 35387989 10.1038/s41467-022-29343-z PMC8987088

[70] Paro R & Hogness DS (1991) The Polycomb protein shares a homologous domain with a heterochromatin‐associated protein of drosophila. Proc Natl Acad Sci USA 88, 263–267. 1898775 10.1073/pnas.88.1.263 PMC50790

[71] Elgin SC & Reuter G (2013) Position‐effect variegation, heterochromatin formation, and gene silencing in drosophila. Cold Spring Harb Perspect Biol 5, a017780. 23906716 10.1101/cshperspect.a017780 PMC3721279

[72] Shermoen AW , McCleland ML & O'Farrell PH (2010) Developmental control of late replication and S phase length. Curr Biol 20, 2067–2077. 21074439 10.1016/j.cub.2010.10.021 PMC3108027

[73] Zhimulev IF (1998) Polytene chromosomes, heterochromatin, and position effect variegation. Adv Genet 37, 1–566. 9352629 10.1016/s0065-2660(08)60341-7

[74] Paro R (1990) Imprinting a determined state into the chromatin of drosophila. Trends Genet 6, 416–421. 1982376 10.1016/0168-9525(90)90303-n

[75] Rao SS , Huntley MH , Durand NC , Stamenova EK , Bochkov ID , Robinson JT , Sanborn AL , Machol I , Omer AD , Lander ES *et al*. (2014) A 3D map of the human genome at kilobase resolution reveals principles of chromatin looping. Cell 159, 1665–1680. 25497547 10.1016/j.cell.2014.11.021 PMC5635824

[76] Dixon JR , Selvaraj S , Yue F , Kim A , Li Y , Shen Y , Hu M , Liu JS & Ren B (2012) Topological domains in mammalian genomes identified by analysis of chromatin interactions. Nature 485, 376–380. 22495300 10.1038/nature11082 PMC3356448

[77] Ogiyama Y , Schuettengruber B , Papadopoulos GL , Chang JM & Cavalli G (2018) Polycomb‐dependent chromatin looping contributes to gene silencing during drosophila development. Mol Cell 71, 73–88. 30008320 10.1016/j.molcel.2018.05.032

[78] Kahn TG , Savitsky M , Kuong C , Jacquier C , Cavalli G , Chang JM & Schwartz YB (2023) Topological screen identifies hundreds of Cp190‐ and CTCF‐dependent drosophila chromatin insulator elements. Sci Adv 9, eade0090. 36735780 10.1126/sciadv.ade0090 PMC9897668

[79] Brown JL , Zhang L , Rocha PP , Kassis JA & Sun MA (2024) Polycomb protein binding and looping in the ON transcriptional state. Sci Adv 10, eadn1837. 38657072 10.1126/sciadv.adn1837 PMC11042752

[80] Eagen KP , Aiden EL & Kornberg RD (2017) Polycomb‐mediated chromatin loops revealed by a subkilobase‐resolution chromatin interaction map. Proc Natl Acad Sci USA 114, 8764–8769. 28765367 10.1073/pnas.1701291114 PMC5565414

[81] Mateo LJ , Murphy SE , Hafner A , Cinquini IS , Walker CA & Boettiger AN (2019) Visualizing DNA folding and RNA in embryos at single‐cell resolution. Nature 568, 49–54. 30886393 10.1038/s41586-019-1035-4 PMC6556380

[82] Szabo Q , Jost D , Chang JM , Cattoni DI , Papadopoulos GL , Bonev B , Sexton T , Gurgo J , Jacquier C , Nollmann M *et al*. (2018) TADs are 3D structural units of higher‐order chromosome organization in drosophila. Sci Adv 4, eaar8082. 29503869 10.1126/sciadv.aar8082 PMC5829972

[83] Murphy SE & Boettiger AN (2024) Polycomb repression of Hox genes involves spatial feedback but not domain compaction or phase transition. Nat Genet 56, 493–504.38361032 10.1038/s41588-024-01661-6

[84] Francis NJ , Kingston RE & Woodcock CL (2004) Chromatin compaction by a polycomb group protein complex. Science 306, 1574–1577. 15567868 10.1126/science.1100576

[85] Cheutin T & Cavalli G (2018) Loss of PRC1 induces higher‐order opening of Hox loci independently of transcription during drosophila embryogenesis. Nat Commun 9, 3898. 30254245 10.1038/s41467-018-05945-4 PMC6156336

[86] Eskeland R , Leeb M , Grimes GR , Kress C , Boyle S , Sproul D , Gilbert N , Fan Y , Skoultchi AI , Wutz A *et al*. (2010) Ring1B compacts chromatin structure and represses gene expression independent of histone ubiquitination. Mol Cell 38, 452–464. 20471950 10.1016/j.molcel.2010.02.032 PMC3132514

[87] Frey F , Sheahan T , Finkl K , Stoehr G , Mann M , Benda C & Muller J (2016) Molecular basis of PRC1 targeting to Polycomb response elements by PhoRC. Genes Dev 30, 1116–1127. 27151979 10.1101/gad.279141.116 PMC4863741

[88] Brangwynne CP , Mitchison TJ & Hyman AA (2011) Active liquid‐like behavior of nucleoli determines their size and shape in Xenopus laevis oocytes. Proc Natl Acad Sci USA 108, 4334–4339. 21368180 10.1073/pnas.1017150108 PMC3060270

[89] Alberti S , Gladfelter A & Mittag T (2019) Considerations and challenges in studying liquid‐liquid phase separation and biomolecular condensates. Cell 176, 419–434. 30682370 10.1016/j.cell.2018.12.035 PMC6445271

[90] McSwiggen DT , Mir M , Darzacq X & Tjian R (2019) Evaluating phase separation in live cells: diagnosis, caveats, and functional consequences. Genes Dev 33, 1619–1634. 31594803 10.1101/gad.331520.119 PMC6942051

[91] Niekamp S , Marr SK , Oei TA , Subramanian R & Kingston RE (2024) Modularity of PRC1 composition and chromatin interaction define condensate properties. Mol Cell 84, 1651–1666. 38521066 10.1016/j.molcel.2024.03.001 PMC11234260

[92] Plys AJ , Davis CP , Kim J , Rizki G , Keenen MM , Marr SK & Kingston RE (2019) Phase separation of Polycomb‐repressive complex 1 is governed by a charged disordered region of CBX2. Genes Dev 33, 799–813. 31171700 10.1101/gad.326488.119 PMC6601514

[93] Tatavosian R , Kent S , Brown K , Yao T , Duc HN , Huynh TN , Zhen CY , Ma B , Wang H & Ren X (2019) Nuclear condensates of the Polycomb protein chromobox 2 (CBX2) assemble through phase separation. J Biol Chem 294, 1451–1463. 30514760 10.1074/jbc.RA118.006620 PMC6364756

[94] Lau MS , Schwartz MG , Kundu S , Savol AJ , Wang PI , Marr SK , Grau DJ , Schorderet P , Sadreyev RI , Tabin CJ *et al*. (2017) Mutation of a nucleosome compaction region disrupts Polycomb‐mediated axial patterning. Science 355, 1081–1084. 28280206 10.1126/science.aah5403 PMC5503153

[95] Brown K , Chew PY , Ingersoll S , Espinosa JR , Aguirre A , Espinoza A , Wen J , Astatike K , Kutateladze TG , Collepardo‐Guevara R *et al*. (2024) Principles of assembly and regulation of condensates of Polycomb repressive complex 1 through phase separation. Cell Rep 43, 113997. 38493477 10.1016/j.celrep.2024.113997 PMC11061859

[96] Uckelmann M , Levina V , Taveneau C , Ng XH , Pandey V , Martinez J , Mendiratta S , Houx J , Boudes M , Venugopal H *et al*. (2025) Dynamic PRC1‐CBX8 stabilizes a porous structure of chromatin condensates. Nat Struct Mol Biol 32, 520–530. 39815045 10.1038/s41594-024-01457-6 PMC11919719

[97] Lizana L , Nahali N & Schwartz YB (2023) Polycomb proteins translate histone methylation to chromatin folding. J Biol Chem 299, 105080. 37499944 10.1016/j.jbc.2023.105080 PMC10470199

[98] Boivin A & Dura JM (1998) In vivo chromatin accessibility correlates with gene silencing in drosophila. Genetics 150, 1539–1549. 9832530 10.1093/genetics/150.4.1539 PMC1460423

[99] Fitzgerald DP & Bender W (2001) Polycomb group repression reduces DNA accessibility. Mol Cell Biol 21, 6585–6597. 11533246 10.1128/MCB.21.19.6585-6597.2001 PMC99804

[100] Schlossherr J , Eggert H , Paro R , Cremer S & Jack RS (1994) Gene inactivation in drosophila mediated by the Polycomb gene product or by position‐effect variegation does not involve major changes in the accessibility of the chromatin fibre. Mol Gen Genet 243, 453–462. 7911223 10.1007/BF00280476

[101] King HW , Fursova NA , Blackledge NP & Klose RJ (2018) Polycomb repressive complex 1 shapes the nucleosome landscape but not accessibility at target genes. Genome Res 28, 1494–1507. 30154222 10.1101/gr.237180.118 PMC6169895

[102] Dunjic M , Jonas F , Yaakov G , More R , Mayshar Y , Rais Y , Orenbuch AH , Cheng S , Barkai N & Stelzer Y (2023) Histone exchange sensors reveal variant specific dynamics in mouse embryonic stem cells. Nat Commun 14, 3791. 37365167 10.1038/s41467-023-39477-3 PMC10293259

[103] Mito Y , Henikoff JG & Henikoff S (2007) Histone replacement marks the boundaries of cis‐regulatory domains. Science 315, 1408–1411. 17347439 10.1126/science.1134004

[104] Dellino GI , Schwartz YB , Farkas G , McCabe D , Elgin SC & Pirrotta V (2004) Polycomb silencing blocks transcription initiation. Mol Cell 13, 887–893. 15053881 10.1016/s1097-2765(04)00128-5

[105] Owen JA , Osmanovic D & Mirny L (2023) Design principles of 3D epigenetic memory systems. Science 382, eadg3053. 37972190 10.1126/science.adg3053 PMC11075759

[106] Coleman RT & Struhl G (2017) Causal role for inheritance of H3K27me3 in maintaining the OFF state of a drosophila HOX gene. Science 356, eaai8236. 28302795 10.1126/science.aai8236 PMC5595140

[107] Laprell F , Finkl K & Muller J (2017) Propagation of Polycomb‐repressed chromatin requires sequence‐specific recruitment to DNA. Science 356, 85–88. 28302792 10.1126/science.aai8266

[108] Lundkvist MJ , Lizana L & Schwartz YB (2023) Forecasting histone methylation by Polycomb complexes with minute‐scale precision. Sci Adv 9, eadj8198. 38134278 10.1126/sciadv.adj8198 PMC10745708

[109] Margueron R , Justin N , Ohno K , Sharpe ML , Son J , Drury WJ 3rd , Voigt P , Martin SR , Taylor WR , De Marco V *et al*. (2009) Role of the polycomb protein EED in the propagation of repressive histone marks. Nature 461, 762–767. 19767730 10.1038/nature08398 PMC3772642

[110] Lee HG , Kahn TG , Simcox A , Schwartz YB & Pirrotta V (2015) Genome‐wide activities of Polycomb complexes control pervasive transcription. Genome Res 25, 1170–1181. 25986499 10.1101/gr.188920.114 PMC4510001

[111] Szczurek AT , Dimitrova E , Kelley JR , Blackledge NP & Klose RJ (2024) The Polycomb system sustains promoters in a deep OFF state by limiting pre‐initiation complex formation to counteract transcription. Nat Cell Biol 26, 1700–1711. 39261718 10.1038/s41556-024-01493-w PMC11469961

[112] Robinson JT , Turner D , Durand NC , Thorvaldsdottir H , Mesirov JP & Aiden EL (2018) Juicebox.Js provides a cloud‐based visualization system for hi‐C data. Cell Syst 6, 256–258. 29428417 10.1016/j.cels.2018.01.001 PMC6047755

